# A Novel Deep Learning Model for Accurate Pest Detection and Edge Computing Deployment

**DOI:** 10.3390/insects14070660

**Published:** 2023-07-24

**Authors:** Huangyi Kang, Luxin Ai, Zengyi Zhen, Baojia Lu, Zhangli Man, Pengyu Yi, Manzhou Li, Li Lin

**Affiliations:** 1 College of Information and Electrical Engineering, China Agricultural University, Beijing 100083, China; vagabond@cau.edu.cn (H.K.); zyz5doc@cau.edu.cn (Z.Z.); pengyuyi@cau.edu.cn (P.Y.); 2 College of Plant Protection, China Agricultural University, Beijing 100083, China; lx_ai@cau.edu.cn (L.A.); zlm@cau.edu.cn (Z.M.); limanzhou_cau@163.com (M.L.); 3 Faculty of Information Technology, Beijing University of Technology, Beijing 100124, China; wkbyg@126.com

**Keywords:** pest detection, deep learning, multi-scale feature fusion, edge computing, knowledge distillation

## Abstract

**Simple Summary:**

This research proposes a novel attention mechanism for the task of rice pest detection, aiming to address the issues of complex backgrounds and small size of pests. By dynamically adjusting attention weights, the model effectively focuses on small-scale pests, avoiding distractions from complex background information. Concurrently, we adopt a multi-scale feature fusion technique, successfully extracting rich and distinctive features, thereby further enhancing the model’s performance. Numerous experiments demonstrate superior performance of our model over advanced methods like YOLO, EfficientDet, RetinaDet, and MobileNet in pest detection tasks. Overall, through innovative attention mechanism and feature fusion techniques, our work effectively tackles the critical issues in pest detection, achieving excellent detection results.

**Abstract:**

In this work, an attention-mechanism-enhanced method based on a single-stage object detection model was proposed and implemented for the problem of rice pest detection. A multi-scale feature fusion network was first constructed to improve the model’s predictive accuracy when dealing with pests of different scales. Attention mechanisms were then introduced to enable the model to focus more on the pest areas in the images, significantly enhancing the model’s performance. Additionally, a small knowledge distillation network was designed for edge computing scenarios, achieving a high inference speed while maintaining a high accuracy. Experimental verification on the IDADP dataset shows that the model outperforms current state-of-the-art object detection models in terms of precision, recall, accuracy, mAP, and FPS. Specifically, a mAP of 87.5% and an FPS value of 56 were achieved, significantly outperforming other comparative models. These results sufficiently demonstrate the effectiveness and superiority of the proposed method.

## 1. Introduction

With the continued growth of the global population, the pressure on the food supply is increasing. As one of the world’s major food crops, the improvement of rice yield has significant implications for ensuring global food security. However, in the process of rice production, pest infestations are one of the main factors affecting yield and quality [1,2]. Pest occurrences are closely related to the formation of gut microbes within the host [3]. Parasites not only manipulate hosts but also effectively utilize them, significantly impacting plants [4]. Traditional pest control methods mainly rely on manual field inspection [5], followed by the selection of appropriate control strategies based on the pest species and density [6]. However, this method is inefficient, time-consuming, labor-intensive, and challenging to implement on a large scale [7].

In recent years, the rapid development of modern information technology, artificial intelligence, and deep learning has achieved significant success in many fields. Particularly in the field of computer vision, deep learning provides solutions for image recognition and object detection. However, despite the excellent performance of deep learning methods in many tasks [8,9,10], there are still some challenges in pest recognition and detection in complex environments. For instance, due to the complexity of the image background and the small size of pests, which are similar in color to the background, there is room for improvement in the accuracy and real-time performance of traditional deep learning models [11].

Therefore, the development of a deep learning model that can accurately and quickly detect rice pests in complex environments is of great practical significance for improving rice production efficiency and ensuring food security [12]. This could not only alleviate farmers’ labor intensity and improve the efficiency and accuracy of pest control but also provide technical support for the development of precision agriculture and smart agriculture [13].

Many researchers have conducted studies in this area. For instance, Yuqi Hu proposed a deep neural network named YOLO-GBS for detecting and classifying pests from digital images. Experimental results showed that the average mAP of the model, which includes Crambidae, Noctuidae, Ephydridae, and Delphacidae, reached as high as 79.8%, 5.4% higher than YOLOv5s, and the detection effect in various complex scenes was significantly improved [14]. Shuai Yang proposed a high-precision and real-time corn pest detection method—Maize-YOLO. This network is based on YOLOv7 and inserts the CSPResNeXt-50 module and VoVGSCSP module. Experimental results showed that the method outperforms the YOLO series object detection algorithm and achieved a 76.3% mAP and a 77.3% recall rate [15]. Tannous developed a detection method based on convolutional neural networks, which can accurately and in real time classify two types of freely moving and changing posture mollusks (Ceratitis capitata and Bactrocera oleae). The results showed an accuracy rate of approximately 93% [16]. Min Dai improved upon a method based on YOLOv5m and proposed a plant pest detection approach with a higher precision. Experimental results indicate that the improved YOLOv5m achieved a precision of 95.7%, a recall of 93.1%, an F1 score of 94.38%, and a mean average precision (mAP) of 96.4%. Additionally, the proposed model significantly outperforms the original YOLOv3, YOLOv4, and YOLOv5m models [17]. Yue Teng put forward a robust aphid detection method, incorporating two customized core designs: a Deformable Feature Pyramid Network (T-FPN) and a Multi-Resolution Training Method (MTM), achieving a mean recall of 46.1% and a mean precision of 74.2%. This surpasses other state-of-the-art methods, including ATSS, Cascade R-CNN, FCOS, FoveaBox, and CRA-Net [18]. Chu-Yuan Luo constructed a tick identification tool that can distinguish the most common human-biting ticks, namely Amblyomma americanum, Dermacentor variabilis, and Ixodes scapularis, by implementing artificial intelligence methods with deep learning algorithms. The best CNN model reached an accuracy of 99.5% on the test set [19]. Mark T. Fowler pretrained a resnet-50 CNN using the ImageNet dataset with TensorFlow. The structure was retrained, achieving an accuracy of 94%, with an average application time of 38.5 s [20]. Qingwen Guo used saliency maps and an improved non-maximum suppression to compute the number of insect pests, achieving a significant improvement in the F1 score [21]. Suk-Ju Hong proposed an automatic deep learning counting algorithm for pheromone trap images, with most models counting accuracies exceeding 95% [22]. Zhiliang Zhang put forward a method for detecting small tyrant grooming behavior based on computer vision and artificial intelligence. The method for detecting small tyrant grooming behavior can save a lot of manpower, with a detection accuracy of over 95% and a difference of less than 15% compared with the results of manual observation [23]. Sijing Ye proposed a method using CNN technology, using the proposed ResNet-Locust-BN model to identify locust species and instances. The model also performed well in identifying the growth status information of AM locusts (three-instar nymphs (accuracy 77.20%), five-instar nymphs (accuracy 88.40%), and adults (accuracy 93.80%)), with an overall accuracy of 90.16% [24].

In the field of rice pest detection research, despite the existence of many studies using attention mechanisms, our work differs from them in several key aspects. First, our attention mechanism is specially designed to address specific issues in pest detection tasks, namely, the complex backgrounds and small size of pests. Our model, by dynamically adjusting attention weights, can focus more effectively on small-sized pests, without being distracted by complex background information. Meanwhile, our attention mechanism operates at the feature level, which also helps to capture pest features at different scales. Second, we have not only theoretically designed this novel attention mechanism but also validated its effectiveness through a large number of experiments. These include ablation studies based on different features and comparisons with other advanced methods (such as YOLO, EfficientDet, RetinaDet, and MobileNet). The results of these experiments consistently demonstrate the superior performance of our model in pest detection tasks.

Another significant innovation of this study is the design of a small knowledge distillation network for edge computing scenarios. Due to the limited computing capabilities and storage space of edge computing devices, designing a lightweight model that maintains a high precision is crucial for practical applications. The small network distills knowledge from the attention-mechanism-enhanced model, significantly reducing the size and computational complexity of the model while maintaining a high accuracy. This makes it more suitable for deployment on edge computing devices.

A series of experiments were conducted to validate the performance of the proposed model. The IDADP dataset, which contains images of six types of rice pests with high resolutions and diverse backgrounds, was used for training and testing. It is ideal for testing the performance of the model in complex environments. Experimental results show that the proposed model outperforms existing models, such as RetinaDet [25], EfficientDet [26], YOLOv5 [27], YOLOv8 [28], FasterRCNN [29], and MaskRCNN [30], in terms of accuracy, recall, precision, mAP, and FPS. Furthermore, ablation experiments were conducted on different attention mechanisms and data augmentation strategies, further verifying the effectiveness of the proposed model and strategies. Notably, the small knowledge distillation network significantly outperforms the large network in inference speed with only a minor loss in accuracy, making the model very suitable for deployment on edge devices with limited computing capabilities. Finally, the main innovative points of this paper are as follows:

1. We proposed a novel attention mechanism that specifically addresses the issues of the complex backgrounds and small size of pests.
2. We used multi-scale feature fusion techniques to effectively extract richer, more distinguishable features, thereby enhancing the performance of the model.
3. Through a large number of ablation experiments and comparisons with other advanced methods, we have validated the effectiveness and superiority of our model.

In summary, our work effectively solves key problems in pest detection by proposing a novel attention mechanism and using multi-scale feature fusion techniques, achieving a superior detection performance.

## 2. Related Work

In this section, a discussion will be presented regarding work pertinent to this paper, including deep learning models extensively employed for object detection tasks. The principles behind these models will be briefly introduced, supplemented by necessary mathematical formulae.

### 2.1. RetinaDet

RetinaDet is a single-stage object detection model based on Focal Loss, which realizes a high detection speed while maintaining accuracy. The pivotal innovation of RetinaDet lies in the introduction of Focal Loss, aiming to solve class imbalance problems. The definition of Focal Loss is as follows:

(1) $$\mathrm{FL}\left(p_{t}\right)=-{\left(1-p_{t}\right)}^{\gamma }log\left(p_{t}\right)$$

Here, $p_{t}$ represents the probability predicted by the model and γ is a hyperparameter to control the degree of attention that the loss function pays to simple and hard samples.

### 2.2. EfficientDet

EfficientDet is a multi-scale feature fusion object detection model based on EfficientNet. Its innovation lies in the introduction of a new network structure, Compound Scaling, for simultaneously optimizing the network depth, width, and input resolution. EfficientDet introduces a new feature fusion module, BiFPN (Bidirectional Feature Pyramid Network), enabling the model to more effectively fuse feature information at different levels and thus enhancing model detection accuracy. The update formula for BiFPN is as follows:

(2) $$F_{i}^{n}=\sum_{j}\frac{w_{j}^{n}F_{j}^{n}}{\sum_{k}w_{k}^{n}}$$

where $F_{i}^{n}$ represents the *i*th layer feature in the *n*th iteration, $w_{j}^{n}$ represents the weight of the *j*th layer feature in the *n*th iteration, and the weights are learned.

### 2.3. YOLOv5

YOLOv5 is a variant of the YOLO series of object detection models. It further optimizes YOLOv4, enhancing the detection speed and accuracy of the model. YOLOv5 makes some improvements in the network structure, such as introducing CIOU loss to replace the original GIoU loss, to more accurately measure the overlap between prediction boxes and actual boxes. The formula for CIOU loss is:

(3) $$CIOU=IOU-\frac{d^{2}}{c^{2}}-\frac{{\left(ar-ap\right)}^{2}}{ar^{2}}$$

where IOU represents the intersection over union of the predicted and actual boxes, *d* is the distance between the centers of the predicted and actual boxes, *c* is the diagonal length of the smallest enclosing rectangle containing the predicted and actual boxes, and ar and ap represent the aspect ratios of the actual and predicted boxes, respectively.

### 2.4. YOLOv8

YOLOv8 is the latest version of the YOLO series of object detection models. It further improves upon YOLOv7, enhancing the model detection accuracy. YOLOv8 makes a series of improvements to the network structure, such as introducing new attention modules and convolution modules to enhance the model’s feature extraction capabilities and receptive field.

### 2.5. Faster R-CNN

Faster R-CNN is an improved version of the R-CNN series models. It introduces a Region Proposal Network (RPN) to the original R-CNN model to accelerate the generation of object candidate regions. The objective function of Faster R-CNN is as follows:

(4) $$L\left(p_{i},t_{i}\right)=\frac{1}{N_{c}ls}\sum L_{cls}\left(p_{i},p_{i}^{*}\right)+\lambda \frac{1}{N_{reg}}\sum_{i}p_{i}^{*}L_{reg}\left(t_{i},t_{i}^{*}\right)$$

Here, $p_{i}$ denotes the probability of the *i*th anchor being an object; $t_{i}$ denotes the coordinates of the *i*th anchor; $p_{i}^{*}$ and $t_{i}^{*}$, respectively, represent the true label and coordinates of the *i*th anchor; and $L_{cls}$, and $L_{reg}$, respectively, represent the classification loss and regression loss.

### 2.6. Mask R-CNN

Mask R-CNN is an extension of Faster R-CNN. It introduces a parallel branch to Faster R-CNN for generating object segmentation masks, enabling Mask R-CNN to achieve pixel-level object segmentation while conducting object detection. The loss function of Mask R-CNN, in addition to the classification loss and regression loss of Faster R-CNN, includes a mask loss:

(5) $$L_{MaskRCNN}=L_{cls}+\lambda_{box}L_{box}+\lambda_{mask}L_{mask}+\lambda_{kp}L_{kp}$$

where $L_{mask}$ represents the mask loss, used to measure the difference between the predicted mask and the actual mask.

These comparative models used in the experiments each have their strengths and weaknesses. Without exception, they have all made significant contributions to the development of object detection tasks. In the following section, an attention mechanism enhancement based on a single-stage object detection model, a multi-scale feature fusion network construction, and a small network design for edge computing scenarios through knowledge distillation will be introduced.

## 3. Materials

This section elucidates the datasets employed in the study, highlighting their characteristics, along with the data augmentation strategy implemented.

### 3.1. IDADP Dataset Analysis

**Figure 1:**
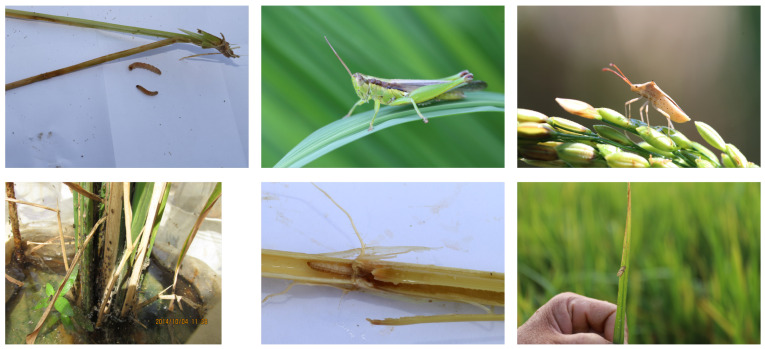
Samples of IDADP dataset.

The IDADP (Insect Detection and Analysis in Digital Pictures) dataset is tailored for insect detection and analysis tasks. It encompasses images of six types of rice pests: Spodoptera litura, Chilo suppressalis, Leptocorisa chinensis, Cnaphalocrocis medinalis, Locusta migratoria manilensis, and Sogatella furcifera, as shown in Figure 1.

Within the IDADP dataset, each image encapsulates one or more types of rice pests, with every pest instance signified by a bounding box and a category label. The bounding box delineates the pest’s location in the image, while the category label indicates the type of pest. Each type of pest is represented in approximately equal quantities of images, resulting in a balanced distribution of categories in the dataset.

The images in the IDADP dataset are of varied resolutions, reaching up to 4 K. These high-resolution images provide rich detail, aiding the model in detecting and categorizing pests more accurately. However, they also demand a higher computational power and processing speed from the model.

### 3.2. Data Augmentation

**Figure 2:**
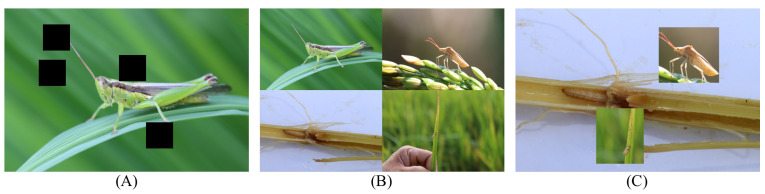
(**A**) is the Cutout; (**B**) is the Mosaic; (**C**) is the Cutmix.

A suite of data augmentation techniques, including Cutout, Cutmix, and Mosaic [31], were utilized in this study to enhance the model’s generalization capability and robustness, as shown in Figure 2.

#### 3.2.1. Cutout

Cutout is a data augmentation strategy that simulates occlusions in images by randomly selecting a region and setting its pixel values to 0, as shown in Figure 2A. The process can be represented by the following equation:

(6) $$X_{cutout}=X\cdot M$$

**Figure 3:**
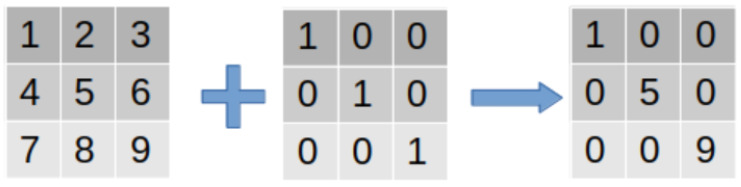
Illustration of a binary mask processing the image.

Here, *X* is the original image and *M* is a binary mask indicating which pixel locations should be set to 0, as shown in Figure 3.

#### 3.2.2. Cutmix

Cutmix is a data augmentation strategy that blends two images, as shown in Figure 2C. It randomly selects a region in the first image and replaces it with the corresponding region from a second image. The process can be represented by the following equation:

(7) $$X_{cutmix}=M\cdot X_{1}+\left(1-M\right)\cdot X_{2}$$

Here, $X_{1}$ and $X_{2}$ are the two original images and *M* is a binary mask indicating the pixel locations originating from $X_{1}$ and $X_{2}$, respectively.

#### 3.2.3. Mosaic

Mosaic is a data augmentation strategy that concatenates four images, as shown in Figure 2B. The images are first scaled to the same size then stitched together in a certain order.

### 3.3. Data Augmentation Using GAN Models

In addition, Generative Adversarial Networks (GANs) were utilized for data augmentation. During the process, a GAN model is trained using the original data, and then this model is used to generate new image data. The GAN model consists of a generator *G* and a discriminator *D*, where the generator *G* attempts to produce fake images indistinguishable from real ones, while the discriminator’s *D* task is to distinguish whether the input image is real or generated by the generator. The training process can be expressed by the following equation:

(8) $$\min_{G}\max_{D}V\left(D,G\right)=Ex∼pdata(x)\left[\log D\left(x\right)\right]+Ez∼pz(z)\left[\log\left(1-D\left(G\left(z\right)\right)\right)\right]$$

Here, *x* represents samples from real data, *z* is the random noise input to the generator, D(x) is the discriminator’s judgment on real images, and D(G(z)) is the discriminator’s judgment on generated images. When generating new image data, noise *z* is first sampled from a preset random distribution and then input to the trained generator to produce new images. This process can be represented as follows:

(9) $$x_{new}=G\left(z\right),z∼p_{z}\left(z\right)$$

Through the above data augmentation strategies and data expansion using GAN models, the diversity of training samples can be significantly increased, thereby enhancing the model’s generalization ability and robustness, which in turn yields improved results in practical applications. The proposed methods will be introduced in the following section.

## 4. Proposed Method

**Figure 4:**
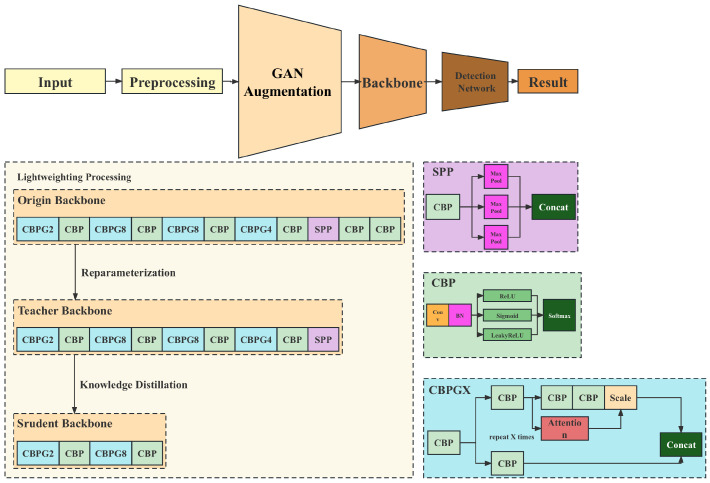
The overview of the proposed method flow.

This section details the method proposed, which is based on a single-stage object detection model. The method primarily comprises three innovative aspects: attention mechanism enhancement based on the single-stage object detection model, the construction of a multi-scale feature fusion network, and designing a small knowledge distillation network tailored for edge computing scenarios. The overview of the proposed method flow is shown in Figure 4.

### 4.1. Attention Mechanism Enhancement Based on Single-Stage Object Detection Model

In the proposed method, an attention mechanism is introduced into the single-stage object detection model, enhancing the model’s focus on targets and thereby improving the precision of object detection. Specifically, an attention module is added to every layer of the feature extraction network. This module can generate an attention map to guide the model to focus more on areas where the target is located.

The specific operation of the attention module can be expressed by the following formula:

(10) A=σ(F∗W+b)

Here, *F* represents the input feature map, *W* and *b* are parameters of the attention module, σ is the sigmoid activation function, and *A* is the generated attention map. After obtaining the attention map, it is multiplied with the original feature map to obtain the enhanced feature map:

(11) $$F^{\prime }=A⊗F$$

Here, ⊗ represents element-wise multiplication. This approach allows the model to focus more on the area where the target is located, thereby improving the precision of object detection.

### 4.2. Construction of Multi-Scale Feature Fusion Network

#### 4.2.1. Significance of Multi-Scale Feature Fusion in Pest Identification

**Figure 5:**
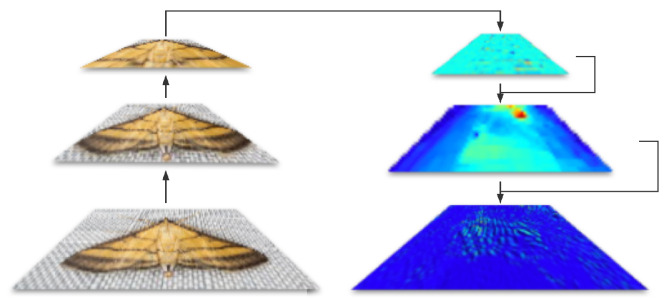
Illustration of multi-scale feature fusion technique in this paper. The left part is the original image and the right part is the feature map generated from original image.

The application of the multi-scale feature fusion technique in this study aims to effectively integrate features from different layers of the network, extracting richer and more distinguishable features to enhance the model performance. This study opted for convolutional neural networks (CNNs) as the baseline model. CNNs possess the ability to extract local features from images. As the network deepens, its feature extraction ability gradually transitions from basic edges and textures to more advanced shapes and parts, as demonstrated in Figure 5.

Therefore, features from different layers can be considered features of different scales. For the task of pest identification, lower-level (shallow) features may include edges, colors, and textures, which play a crucial role in identifying the species and physiological states of pests (such as larvae or adults). In contrast, higher-level (deep) features can extract the overall shape, size, and other global information about the pest, aiding in distinguishing different pest species. Therefore, effectively integrating these features of different layers and scales allows the model to obtain richer and more comprehensive pest feature information. In our task, multi-scale feature fusion played a significant role in two aspects:

1. Pest morphological features exhibit different characteristics on different scales. For instance, at the macro level, we can observe the overall shape, color, and texture of the pest, which are crucial for distinguishing different pest species. On the micro level, we can observe some details of the pest’s body, such as the shape and texture of scales, antennae, wings, etc. These features assist us in more accurately identifying pests.
2. Pests exhibit significant variations in their morphological features at different stages of growth. For example, the shape, size, and color of a pest’s larvae and adult form may differ completely. This necessitates our model’s capability to adapt to such changes and capture the features of pests at different stages. Through multi-scale feature fusion, our model can acquire pest features at different scales, thereby better adapting to the morphological changes of pests and improving identification accuracy.

#### 4.2.2. Implementation of Multi-Scale Feature Fusion

As discussed above, a multi-scale feature fusion network is built to extract and fuse features of different scales. Specifically, convolution operations with kernels of different scales are first performed on the input image, generating a series of feature maps of different scales. These feature maps are then fused through a series of upsampling and downsampling operations to obtain the final feature map. In this task, a multi-scale feature fusion network (MSFFN) is utilized to fully use the information of the target on different scales, thereby improving the performance of the model:

1. Network structure: The structure of the MSFFN mainly includes a backbone network and multiple feature fusion modules.
2. Backbone network: EfficientNet is chosen as the backbone network, which can provide rich and multi-scale feature maps. EfficientNet achieves a high performance while maintaining a low complexity through balanced expansion in network depth, width, and resolution. In this task, EfficientNet-B0 is chosen as the backbone network.
3. Feature fusion module: The feature fusion module mainly includes convolution layers, upsampling layers, and downsampling layers. These layers are used for the fusion and processing of feature maps of different scales. Specifically, convolution layers are first used to extract local information from feature maps, then the scale of the feature maps is adjusted to be consistent through upsampling and downsampling operations, and, finally, these feature maps are fused. The fusion process of features can be represented by the following mathematical formula:
  (12) $$F_{i}=Conv\left(Up\left(F_{i+1}\right)\right)⊕Conv\left(Down\left(F_{i-1}\right)\right)⊕Conv\left(F_{i}\right)$$
  Here, $F_{i}$ is the feature map of the *i*th layer; Up and Down represent upsampling and downsampling operations, respectively; Conv represents the convolution operation; and ⊕ represents the fusion of feature maps, which can be an addition or concatenation operation.
4. Channel number of the feature map: In the MSFFN, the number of channels in the feature map is predominantly adjusted through the convolution layer. To be specific, a convolution layer is implemented in each feature fusion module to adjust the number of channels in the feature map. This adjustment enables the maintenance of a consistent number of channels whilst fusing the feature maps.

Through such a design, the MSFFN can fully utilize the object’s information at different scales, thereby enhancing the model’s performance.

### 4.3. Design of a Small Network for Edge Computing Scenarios Using Knowledge Distillation

In edge computing scenarios, due to hardware resource limitations, there is a need to design a lightweight model for object detection. Therefore, a method of knowledge distillation is introduced, allowing the large model to transfer knowledge to the small model. This transfer facilitates the small model in maintaining a high accuracy while meeting the requirements of edge computing. The knowledge distillation process consists of two stages: initially, a large model (also known as the teacher model) is trained, followed by the training of a small model (also known as the student model). During the training process, the student model learns not only the label information of the data but also the prediction results of the teacher model. The loss function of knowledge distillation can be expressed by the following formula:

(13) $$L=L_{CE}+\alpha L_{KD}$$

Here, $L_{CE}$ represents the cross-entropy loss of the student model, $L_{KD}$ denotes the loss of knowledge distillation, and α is a balance coefficient. $L_{KD}$ can be calculated using the following formula:

(14) $$L_{KD}=T^{2}KL\left(\frac{S}{T},\frac{T}{T}\right)$$

Here, *S* is the prediction result of the student model, *T* is the prediction result of the teacher model, and KL is the Kullback–Leibler divergence used to measure the similarity between two distributions.

**Figure 6:**
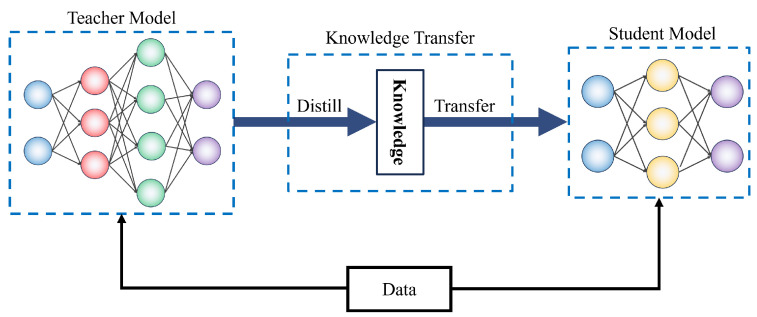
Illustration of knowledge distillation.

Through this method, the small model can learn the knowledge of the large model, thereby achieving efficient object detection in edge computing scenarios. In this task, the process of knowledge distillation primarily consists of two steps: first, training a large network (teacher network) and then using the output of this network to guide the training of the small network (student network), as shown in Figure 6.

The training processes are as follows:

1. Training of the teacher network: The teacher network is typically a large, deep network, such as the single-stage object detection model enhanced by the attention mechanism in this study. This network can have more parameters and a deeper network structure during training, thus acquiring more features and information. Then, the IDADP dataset is used to train this large network to optimize its performance in the object detection task.
2. Training of the student network: The student network is typically a smaller, shallow network. Its purpose is to maintain a high performance while reducing the computation and storage requirements. When training the student network, not only is the standard loss function (such as cross-entropy loss) used but also the output of the teacher network, which is used as a “soft label” to guide the training of the student network. Specifically, the KL divergence between the output of the teacher network and the student network is calculated as an additional loss to force the student network to mimic the behavior of the teacher network. This additional loss function can be expressed as follows:
  (15) $$L_{KD}=\alpha L_{CE}+\left(1-\alpha \right)T^{2}KL\left(\frac{\mathrm{Softmax}(z_{s}/T)}{\mathrm{Softmax}(z_{t}/T)}\right)$$
  Here, $L_{CE}$ denotes the cross-entropy loss; $z_{s}$ and $z_{t}$ represent the logits of the student network and the teacher network, respectively; *T* is a temperature parameter; and α is a weight parameter used to balance the cross-entropy loss and the KL divergence loss.

In this way, the student network not only learns the true labels of the data but also learns the behavior of the teacher network, thereby achieving the goal of maintaining a high detection performance while reducing the model size.

### 4.4. Experiment Settings

For the experiments, a server equipped with an Nvidia Tesla V100 GPU and an Intel(R) Xeon(R) CPU E5-2698 v4 @ 2.20 GHz was used for training and testing. The server runs on the Ubuntu 18.04.3 LTS operating system. From the software perspective, Python 3.7.10 was used as the language for code development and execution, PyTorch 1.8.1 was the primary deep learning framework, OpenCV 4.5.2 was used for image processing, and other libraries, such as numpy 1.20.1 and scikit-learn 0.24.1, were used for data processing and model evaluation. All the code was developed and run in this hardware and software environment.

The model training employed the Adam optimizer with an initial learning rate set at 0.001. A learning rate decay strategy was implemented, reducing the learning rate to 10% of the original rate every 20 epochs. The model was trained for a total of 100 epochs. The batch size was set to 32, determined by the GPU memory capacity of the hardware platform. Models in comparative experiments, including RetinaDet, EfficientDet, YOLOv5, YOLOv8, FasterRCNN, and MaskRCNN, were all of the latest versions and were set with respective default parameters according to their official documentation. All models were trained and tested under the same environment and datasets to ensure experimental fairness.

### 4.5. Experiment Metric

The experiments employed performance metrics, such as precision, recall, accuracy, mean average precision (mAP), and frames per second (FPS), to evaluate the performance of our model and other comparative models:

1. Precision: Precision refers to the ratio of true positives in the detected positives. Its formula is $precision=\frac{TP}{TP+FP}$, where TP represents true positives, i.e., the number of targets correctly detected by the model, and FP represents false positives, i.e., the number of targets incorrectly detected by the model.
2. Recall: Recall refers to the proportion of actual positives detected. Its formula is $recall=\frac{TP}{TP+FN}$, where FN represents false negatives, i.e., the number of actual targets not detected by the model.
3. Accuracy: Accuracy refers to the proportion of all samples (positive and negative) correctly classified. Its formula is $accuracy=\frac{TP+TN}{TP+FP+TN+FN}$, where TN represents true negatives, i.e., the number of non-targets correctly judged by the model.
4. Mean average precision (mAP): mAP is the average precision across all classes, an indicator that takes into account both precision and recall. In object detection, the AP of each class is obtained by calculating the area under the precision–recall curve.
5. Frames per second (FPS): FPS is a critical indicator of model speed, denoting the number of frames the model can process per second. A higher value for the FPS implies a faster detection speed of the model.

These metrics were chosen as they evaluate the model’s performance from various perspectives, including the model’s precision, recall ability, overall performance, and running speed, etc. They allow a comprehensive understanding of the performance of our model and other comparative models in the task.

## 5. Results

### 5.1. Pest Detection Results

In this section, a detailed evaluation of the proposed model and several state-of-the-art object detection models, including RetinaDet, EfficientDet, YOLOv5, YOLOv8, FasterRCNN, and MaskRCNN, will be reported. All models were trained and tested on the IDADP dataset, and evaluation metrics such as precision, recall, accuracy, mAP, and FPS were used. The experimental results are shown in Table 1.

From the table, it can be observed that all models perform well in terms of precision, recall, accuracy, and mAP, but there are significant differences in frames per second (FPS), indicating variations in processing speed among the models. RetinaDet and EfficientDet exhibit similar accuracies, but EfficientDet achieves a slightly higher mAP, likely due to its more complex model structure and more effective feature extraction capabilities. However, both models have lower FPS scores, which can be attributed to their complex structures that require longer computation times. YOLOv5 and YOLOv8 outperform RetinaDet and EfficientDet in all evaluation metrics, possibly due to the lightweight design of the YOLO series models, which strike a better balance between detection accuracy and speed. Particularly, YOLOv8 performs close to our model in terms of mAP, although with a slightly lower FPS. FasterRCNN and MaskRCNN show relatively poorer performances. While they exhibit comparable precision and recall, their mAP and FPS are lower. This may be attributed to the complexity of these models, which demand significant computational resources and result in slower processing speeds on our hardware.

**Figure 7:**
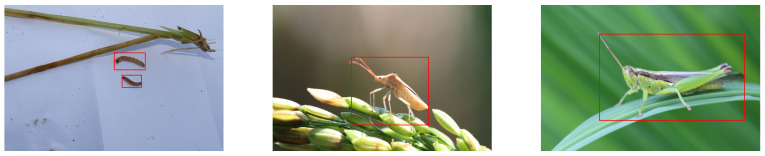
Detection results by proposed method on IDADP dataset. The red boxes are the detection results generated from our method.

The proposed model outperforms all the compared models in all evaluation metrics, as shown in Figure 7, likely owing to our three innovations: attention mechanism enhancement based on a single-stage object detection model, multi-scale feature fusion network construction, and the design of a small knowledge distillation tailored for edge computing scenarios. By introducing the attention mechanism, our model can more accurately focus on regions containing the target, thereby improving detection accuracy. Our multi-scale feature fusion network better utilizes features from different scales, further enhancing the detection performance. Through knowledge distillation, we design a small network that significantly improves the detection speed while ensuring detection accuracy, resulting in a superior FPS performance compared to all the comparative models.

### 5.2. Ablation Study

#### 5.2.1. Ablation Study on Attention Mechanism

In this section, we present some ablation experiments to further validate the effectiveness of our proposed methods. These experiments include ablation experiments on different attention mechanisms, performance tests on different combinations of data augmentation, and inference speed and accuracy tests on the small knowledge distillation network.

Firstly, we conducted ablation experiments on different attention mechanisms. We compared the performance of models without an attention mechanism, with an SE attention mechanism, with a CBAM attention mechanism, and with our proposed attention mechanism. The experimental results are shown in Table 2.

From the ablation experiments on different attention mechanisms, we can observe that both the SE and CBAM attention mechanisms perform better than the model without an attention mechanism. This indicates that attention mechanisms can indeed improve the model’s focus, thereby enhancing the detection performance. Moreover, our proposed attention mechanism outperforms the SE and CBAM mechanisms in terms of performance, indicating that our attention mechanism better utilizes contextual information and effectively focuses on the target.

#### 5.2.2. Ablation Study on Data Augmentation

Next, we performed performance tests on different combinations of data augmentation methods. We compared the performance of models without data augmentation, with Cutout, with Cutmix, with Mosaic, and with GAN-based data augmentation. The experimental results are shown in Table 3.

From the performance tests on different combinations of data augmentation, we can see that all data augmentation methods (Cutout, Cutmix, Mosaic, and GAN-based augmentation) outperform the model without data augmentation. This indicates that data augmentation can effectively increase the model’s robustness and improve detection performance. Among the tested methods, GAN-based data augmentation achieves the best performance, likely because GAN can generate more diverse data, further enhancing the model’s robustness.

#### 5.2.3. Ablation Study on Multi-Scale Feature Fusion

In designing the ablation study on feature multi-scale fusion, we separated the features being fused according to the level or scale and conducted experiments independently. We observed the individual effects of each scale’s features, as well as the effect after their fusion. Through such an ablation study, we can understand the impact of each layer’s features and multi-scale feature fusion on model performance, for example, the performance of shallow features, mid-level features, and deep features individually, as well as their performance when combined in pairs and when fused all together. This will help us understand the contribution of multi-scale feature fusion to model performance enhancement and guide us to further optimize model design and training strategies. The experimental results are shown in Table 4.

According to the results of the ablation study in Table 4, we can clearly see the importance of feature multi-scale fusion in improving the performance of the model. First, it is evident that, whether using shallow features, mid-level features, or deep features, the performance of a model using any single type of features cannot match the performance of a model using all the features. This indicates that, in the pest recognition task, shallow, mid-level, and deep features all have their unique importance and are indispensable. However, from the experiment results of using shallow, mid-level, and deep features separately, the performance of the deep features model is the best, followed by the mid-level features, and the shallow features perform the worst. This suggests that, in this task, deep features (such as the overall shape, size, and other global information of pests) are crucial for pest recognition. However, this does not mean that shallow features and mid-level features are not important. As we can see, the performance of the model improves when we combine shallow features or mid-level features with deep features. This indicates that shallow features and mid-level features can provide some information that deep features cannot obtain, such as edges, color, and texture. Finally, we see that we obtain the best model performance when we combine shallow, mid-level, and deep features. This further verifies the importance of feature multi-scale fusion, i.e., combining features of multiple scales can make the model obtain more abundant and provide comprehensive information, thereby improving the performance of the model. In summary, this ablation study clearly demonstrates the important role of feature multi-scale fusion in the pest recognition task, verifying the effectiveness of our approach.

#### 5.2.4. Ablation Study on Knowledge Distillation

Finally, we conducted inference speed and accuracy tests on the small knowledge distillation network. We compared the performance and inference speed of the original model with the small distilled network. The experimental results are shown in Table 5.

The aforementioned experiment aimed to transfer knowledge from a large deep learning model to a smaller one. The data in Table 5 clearly reveal the outcome of this process. As we can see, the parameter count of the original model was 86.0 M, but the parameter count of the small model after knowledge distillation is only 2.28 M, significantly reducing the model’s size. Similarly, the computational complexity was reduced from 35.1 G to 0.19 G. However, at the same time, metrics such as the precision, recall, and mAP have only seen slight declines. This indicates that, despite a significant reduction in the complexity of the small model, its performance on the pest detection task remains robust. The experimental results can be explained from the following perspectives:

1. Soft labels: In the process of knowledge distillation, the teacher model provides a probability distribution for each class, known as soft labels, instead of single hard labels. Compared to hard labels, soft labels provide more detailed information, helping the student model to learn finer class distinction information.
2. Model capacity: Although the smaller model has fewer parameters, it does not mean it cannot achieve a good performance. In fact, if the amount of data are limited, overly complex models can cause overfitting and therefore cannot achieve a good generalization ability. Through knowledge distillation, we can find a balance point, ensuring the model is not overly complex or overly simplified and thus obtaining an optimal performance.
3. Attention transfer: In some cases, the teacher model may over-attend to unnecessary information and overlook features that are more important to the target task. Through knowledge distillation, the student model can learn these important features from the teacher model, thereby improving its performance.

In summary, the key to knowledge distillation is to leverage the knowledge of the teacher model to aid the student model in learning. This ensures that a high model performance can still be maintained even with a significant reduction in the model parameters and computational complexity. Finally, we can observe that, although the performance of the distilled small model is slightly lower than the original model, its inference speed is significantly improved. This indicates that, through knowledge distillation, we can design a model that maintains a relatively high detection accuracy while achieving a higher inference speed, which is of practical value for real-time pest detection tasks.

Compared with MobileNet, these ablation experiment results demonstrate that our proposed attention mechanism, data augmentation strategies, and knowledge distillation method contribute significantly to the performance improvement of the model. In conclusion, our proposed methods are effective, enabling the model to achieve a high detection accuracy while maintaining a high detection speed.

### 5.3. Exploration of Attention Focus Visualization

To further investigate the impact of the attention mechanism on the performance improvement of our model, we conducted visualizations of the attention maps. By visualizing the attention maps, we can intuitively observe the distribution of the model’s attention on different regions of the input image, understand how the model works, and further demonstrate the effectiveness of our proposed model.

Firstly, it is necessary to understand the role of the attention mechanism in the model. The essence of the attention mechanism is a weight allocation strategy, where different parts of the input are assigned different weights based on their importance. In visual tasks, the attention mechanism often manifests as the model’s focus on certain regions of the image. Specifically, if the model considers a particular region crucial for the task, that region will have a higher weight and the model will pay more attention to it.

**Figure 8:**
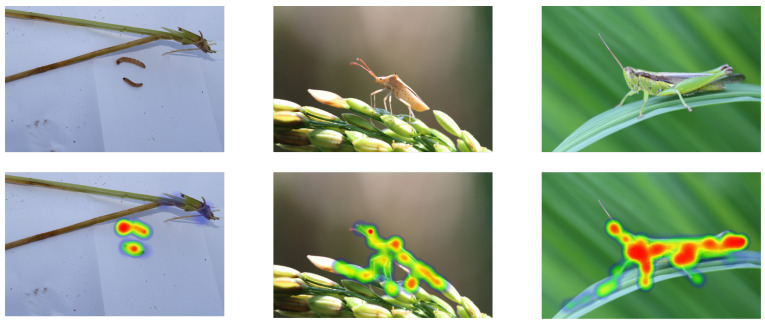
Visualization of the attention mechanism. These red regions highlight the areas that the model deems most relevant or significant for its predictions. Conversely, the areas marked in green represent regions of low attention from the model.

In our task, we employ attention mechanism enhancement based on a single-stage object detection model. This attention mechanism automatically identifies the most important regions in the image for the task, namely, the locations of pests, assisting the model in accurately detecting these pests. During the visualization process, we present the attention distribution of the model on the image by creating heatmaps. The heatmap is a two-dimensional data visualization method that represents data magnitude through variations in color intensity. In our task, the color intensity in the heatmap represents the model’s attention level on different regions of the image. The darker the color, the higher the model’s attention and vice versa, as shown in Figure 8.

From our heatmaps, we can clearly see that the model’s attention is mainly focused on the regions containing pests. This aligns with our expectations because pests are the most important targets in our task, and the model should primarily focus on these regions. This result indicates that our model effectively utilizes the attention mechanism to automatically identify important regions in the image, thereby improving the detection accuracy.

Additionally, we notice that the model also has some level of attention on non-pest regions. This may be due to the presence of features in these regions that resemble pests or due to a data imbalance. Despite this, overall, the attention distribution of the model still aligns with our expectations.

In summary, through the visualization of the attention mechanism, we can gain insight into the model’s focus and observe that the model’s attention is mainly concentrated on the pest regions in the image. This result verifies the effectiveness of our attention mechanism based on a single-stage object detection model.

## 6. Conclusions

Despite the outstanding performance demonstrated in the task of rice pest detection, there are certain limitations recognized and areas identified for further research and improvement.

The proposed model has shown superior performance on the IDADP dataset compared to current state-of-the-art object detection models across various evaluation metrics, such as precision, recall, accuracy, mAP, and FPS. More specifically, a mAP of 87.5% and an FPS value of 56 were achieved, significantly outperforming other comparative models.

However, the model was primarily focused on the pest areas of the images, as the primary task was pest detection. Practical applications may require the detection of other objects, such as leaf blight or the growth state of the rice. These targets may have different features in the images than pests; hence, adjustments to the model may be necessary to cater to these new tasks. Future work will explore how the model can be extended to more object detection tasks.

It was also noticed that the model showed some degree of attention to non-pest areas, possibly due to these areas containing features similar to pests or caused by a dataset imbalance. This issue could be mitigated by data augmentation methods to increase the dataset diversity and further optimize the model performance.

Although attention mechanisms based on single-stage object detection models were used, there is room for improvement. For instance, the introduction of more complex attention mechanisms, such as self-attention mechanisms, could further enhance the model performance. Moreover, the combination of attention mechanisms with other machine learning technologies, such as deep learning or reinforcement learning, could be explored to enhance the model’s performance.

Finally, the model was trained and tested on a specific hardware platform and software environment. However, practical applications may require the model to be used in different hardware platforms and software environments. Thus, optimization of the model for it to maintain a good performance in various environments is necessary.

In future work, the aforementioned issues will be thoroughly researched and improved upon. It is hoped that, through continuous optimization and improvement, the model will play a greater role in more scenarios and tasks.

## Figures and Tables

**Table 1:** Performance of different models on the IDADP dataset.

Model	Precision	Recall	Accuracy	mAP	FPS
RetinaDet [25]	0.80	0.82	0.81	0.79	20
EfficientDet [26]	0.83	0.85	0.84	0.82	18
YOLOv5 [27]	0.86	0.87	0.86	0.85	22
YOLOv8 [28]	0.89	0.90	0.89	0.88	21
FasterRCNN [29]	0.82	0.84	0.83	0.81	16
MaskRCNN [30]	0.81	0.83	0.82	0.80	15
Proposed Model	0.90	0.91	0.91	0.89	56

**Table 2:** Results of ablation experiments on different attention mechanisms.

Attention Mechanism	Precision	Recall	mAP
None	0.88	0.89	0.87
SE [32]	0.89	0.90	0.88
CBAM [33]	0.90	0.91	0.89
Proposed Method	0.92	0.93	0.91

**Table 3:** Performance test results on different combinations of data augmentation.

Data Augmentation	Precision	Recall	mAP
None	0.88	0.89	0.87
Cutout	0.89	0.90	0.88
Cutmix	0.90	0.91	0.89
Mosaic	0.91	0.92	0.90
GAN	0.92	0.93	0.91

**Table 4:** Ablation study results on the feature multi-scale fusion.

Experiment	Precision	Recall	mAP
Shallow Features Only	0.80	0.81	0.78
Mid-level Features Only	0.85	0.86	0.82
Deep Features Only	0.88	0.89	0.87
Shallow + Mid-level Features	0.88	0.88	0.86
Shallow + Deep Features	0.90	0.91	0.88
Mid-level + Deep Features	0.91	0.92	0.90
Shallow + Mid-level + Deep Features	0.92	0.93	0.91

**Table 5:** Results of inference speed and accuracy tests on the small knowledge distillation network.

Model	Parameters	FLOPS	Precision	Recall	mAP	FPS
Original Model	86.0 M	35.1 G	0.92	0.93	0.91	24
Distilled Small Model	2.28 M	0.19 G	0.90	0.91	0.89	56
MobileNet [34]	2.54 M	0.08 G	0.87	0.83	0.85	71

## Data Availability

Data is available on request.
